# Potential of active transport to improve health, reduce healthcare costs, and reduce greenhouse gas emissions: A modelling study

**DOI:** 10.1371/journal.pone.0219316

**Published:** 2019-07-17

**Authors:** Anja Mizdrak, Tony Blakely, Christine L. Cleghorn, Linda J. Cobiac

**Affiliations:** 1 Burden of Disease Epidemiology, Equity, and Cost-Effectiveness Programme, Department of Public Health, University of Otago (Wellington), Wellington, New Zealand; 2 Population Interventions Unit, Centre for Epidemiology and Biostatistics, Melbourne School of Population and Global Health, University of Melbourne, Melbourne, Australia; 3 Centre for Population Approaches to Non-Communicable Disease Prevention, Nuffield Department of Population Health, University of Oxford, Oxford, United Kingdom; Icahn School of Medicine at Mount Sinai, UNITED STATES

## Abstract

**Background:**

Physical inactivity contributes substantively to disease burden, especially in highly car dependent countries such as New Zealand (NZ). We aimed to quantify the future health gain, health-sector cost-savings, and change in greenhouse gas emissions that could be achieved by switching short vehicle trips to walking and cycling in New Zealand.

**Methods:**

We used unit-level survey data to estimate changes in physical activity, distance travelled by mode, and air pollution for: (a) switching car trips under 1km to walking and (b) switching car trips under 5km to a mix of walking and cycling. We modelled uptake levels of 25%, 50%, and 100%, and assumed changes in transport behaviour were permanent. We then used multi-state life table modelling to quantify health impacts as quality adjusted life years (QALYs) gained and changes in health system costs over the rest of the life course of the NZ population alive in 2011 (n = 4.4 million), with 3% discounting.

**Findings:**

The modelled scenarios resulted in health gains between 1.61 (95% uncertainty interval (UI) 1.35 to 1.89) and 25.43 (UI 20.20 to 30.58) QALYs/1000 people, with total QALYs up to 112,020 (UI 88,969 to 134,725) over the remaining lifespan. Healthcare cost savings ranged between NZ$127million (UI $101m to 157m) and NZ$2.1billion (UI $1.6b to 2.6b). Greenhouse gas emissions were reduced by up to 194kgCO_2_e/year, though changes in emissions were not significant under the walking scenario.

**Conclusions:**

Substantial health gains and healthcare cost savings could be achieved by switching short car trips to walking and cycling. Implementing infrastructural improvements and interventions to encourage walking and cycling is likely to be a cost-effective way to improve population health, and may also reduce greenhouse gas emissions.

## Introduction

Transport has a major impact on population health–it directly affects injury rates and air pollution, and indirectly influences physical activity and health impacts arising from climate change. Reducing car use and increasing active transport is expected to improve health at the city-level, nationally, and internationally [1–7].

Setting and population-specific estimates of the impact of transport changes are required to trade-off the positive and negative impacts of increased active transport. For example, there are concerns that increased injury risk or increased air pollution exposure may outweigh the benefits of increased physical activity levels in selected population groups or settings [5].

New Zealand (NZ) is highly car dependent: 79% of all self-reported trips are made by car [8] and car ownership rates are among the highest in the world. Low physical activity is the 4^th^ leading behavioural risk factor in NZ [9], and only half of adults meet the national physical activity recommendations [10]. In addition, 17.3% of gross greenhouse gas emissions in NZ are related to road transport [11].

Internationally, estimates of the health impact of increases in active transport are commonly conducted using comparative risk assessment (CRA) methods [1–4]. However, comparative risk assessment methods do not account for time lags between exposure and disease and long-term changes in survival [12] and this suggests that many studies modelling the health impact of increasing active transport overestimate the benefits.

Previous NZ research based on comparative risk assessment methods strongly suggests that increasing active transport is likely to have positive health impacts and reduce greenhouse gas emissions [4, 7]. However, neither of the previous studies included time lags, estimated the uncertainty around modelled health impacts, nor used a lifetime approach to assess the long-term health impact. These methodological limitations mean it is unclear how the health impact of increasing active transport might compare to addressing other population level risks (e.g. smoking and unhealthy diets). This is particularly problematic from a policy perspective–if the likely impacts of addressing different health risks cannot be compared due to differing methodological approaches then it is difficult to prioritise resource spending appropriately across competing health priorities.

We conducted this study to estimate the health impact, change in health system costs, and greenhouse gas emissions associated with increasing active transport in New Zealand. We use an established multi-state life table modelling approach that has been used to estimate the impact of a wide range of other public health interventions internationally including dietary change, alcohol reduction, and tobacco end game strategies [13–15]. Use of comparable methods facilitates comparison of increasing active transport with interventions addressing other population level risks (e.g. smoking and unhealthy diet).

The aims of this study were: (i) to estimate health impact of switching short trips to walking and cycling; (ii) to estimate change in health system costs associated with modelled changes in transport patterns; (iii) to estimate change in greenhouse gas emissions associated with changes in transport patterns in NZ.

## Methods

### Overview

**Fig 1:**
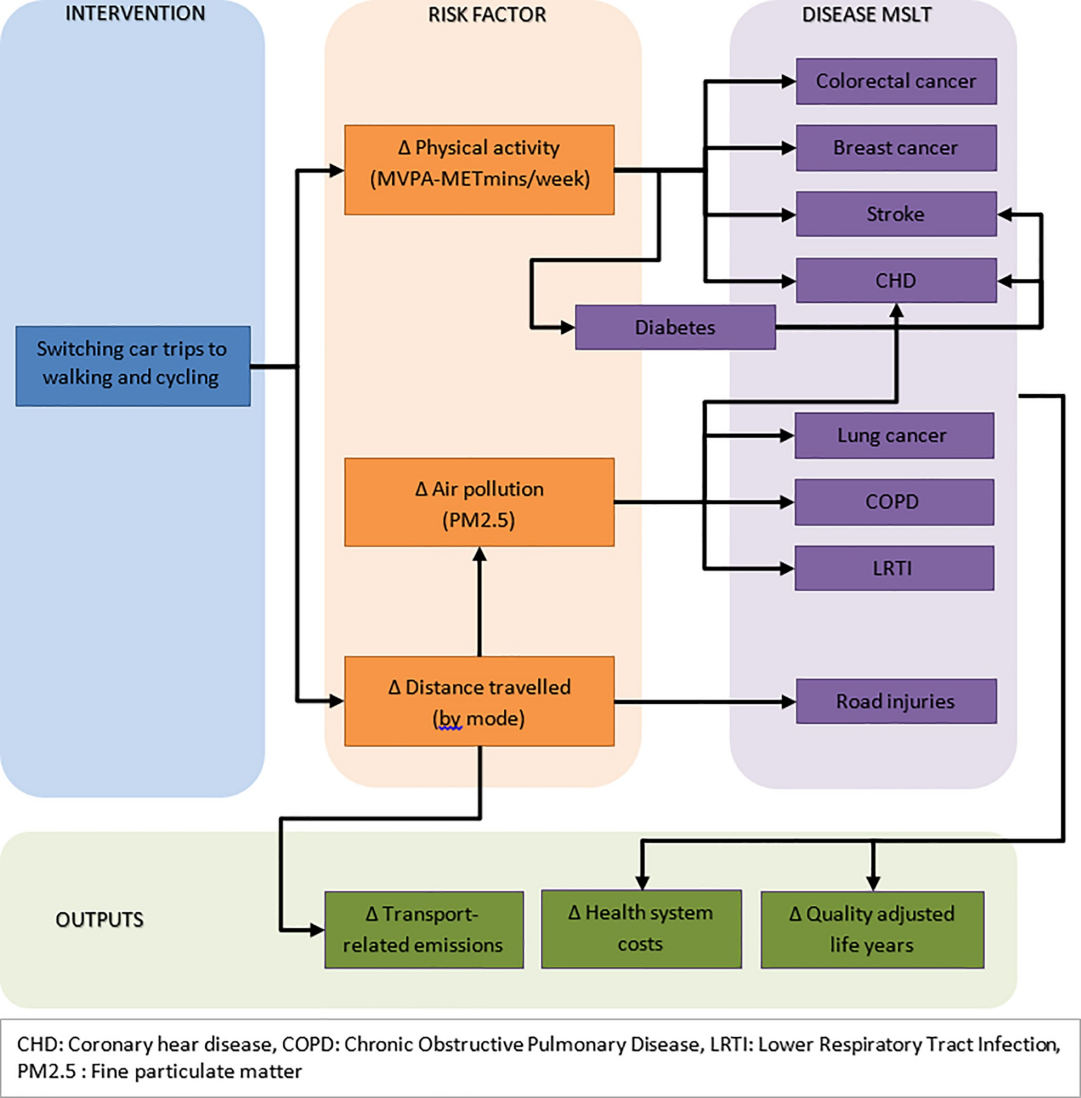
Conceptual framework of the model.

We modelled the differences in quality-adjusted life years (QALYs) and healthcare costs of shifts in transport behaviours in NZ. We estimated changes in transport patterns and greenhouse gas emissions using unit-level survey data and then multi-state life table (MSLT) modelling to determine long-term impacts on population health and change in health system costs. The MSLT part of the model used the intervention impacts (change in physical activity, distance travelled, and air pollution) to estimate impact on QALYs and healthcare costs over the remainder of the lifetime of the New Zealand population alive in 2011. Input parameters included risk factor distributions (mode-specific distance travelled, physical activity, and air pollution) and disease data (including incidence, prevalence, and mortality rates, trends, disability weights, and costs). Sources of model inputs are summarised in Table 1, a conceptual framework of the model structure is outlined in Fig 1, and full model details including parameter values are detailed in a Technical Report [16].

**Table 1:** Description of model inputs.

Input parameter(s)	Detail	Data source
Risk factor		
Physical activity	Minutes per week of moderate and vigorous physical activity (MVPA-METmins/week), weighted by MET value associated with activity. Heterogeneity by age, sex, and ethnicity.	New Zealand Health Survey 2011/12 Compendium of Physical Activities [17]
Distance travelled	Mode-specific total annual distance travelled (for pedestrians, cyclists, motorcyclists, and motor vehicles). Heterogeneity by age, sex, and ethnicity.	New Zealand Household Travel Survey 2003–2014 [18, 19]
Air pollution	Population-weighted annual fine particulate matter exposure (<2.5μm diameter). No heterogeneity.	Brauer et al [20]
Disease and injury parameters		
Disease incidence, prevalence, case-fatality, and mortality rates	Each parameter was first estimated from linked health data, then simultaneously entered into DisMod II (an epidemiological calculator) to ensure coherence. Heterogeneity by age, sex, and ethnicity.	As per Cleghorn et al [21], with model inputs available at [22]
Injury incidence and mortality rates	Derived using GBD data on mode-specific incidence and mortality rates by age and sex, combined with Health Tracker data and NZBDS to estimate rates by ethnicity.	GBD Results Tool [23], NZBDS [24] and Health Tracker
Morbidity rates	In the main lifetable that simulated the QALYs, morbidity for each sex by ethnic by age group in BAU uses the years of life lived with disability (YLD) due to all causes from NZBDS, divided by the number of people in this strata to give a rate. This represents the average ‘background’ morbidity rate experienced. Disease-specific morbidity (or ‘disability’) rates are derived similarly, using disease specific YLDs from the NZBDS. Disease specific morbidity rates reflect the average disability experienced by someone with that specific disease. For example, CHD morbidity rate for 55–64 year old non-Māori males was calculated as follows. NZBDS pYLD estimate for 2006 (1,321) was scaled to account for demographic change to estimate pYLD value for 2011 (1,533). The scaled pYLD estimate was divided by the number of prevalent cases estimated from DisModII (17,326), to give a morbidity rate of 0.088.	GBD [25], NZBDS [24], as per methods described in detail in [21] and [26], with model inputs available at [22]
Healthcare costs (2011 NZ$)	The costs used represent excess annual health system costs for cases in first year of diagnosis, last year of life if dying of that disease, and otherwise prevalent years of diagnosis. Heterogeneity by age and sex, but not ethnicity.	As per Kvizhinadze et al [27], available in [16]

GBD: Global Burden of Disease Study

MET: Metabolic equivalent of task

MVPA: Moderate and vigorous physical activity

NZBDS: New Zealand Burden of Disease Study

YLD: Years lived with disability

See Technical Report [16] for further details on parameters, including uncertainty distributions.

### Intervention scenarios

We modelled the following interventions: (a) switching car trips ≤1km to walking; (b) switching car trips ≤1km to walking and those 1-5km to cycling. In all cases, we switched “there-and-back” trip pairs for individuals aged 15–79 years. We defined there-and-back trip pairs as two consecutive trips, travelling from a start location to a destination and back, using the same mode. Individuals aged 80+ years were excluded from trip switches due to small numbers participating in active transport and high prevalence of co-morbid conditions that could limit participation at older ages.

The NZ Household Travel Survey dataset was used as the baseline population to estimate changes in travel behaviour under the interventions. An anonymised and de-identified dataset was obtained under a confidentiality deed from the New Zealand Ministry of Transport. For each model run, we sampled there-and-back trips within the dataset to switch from driving (driver or passenger) to walking and/or cycling (random sampling based on scenario percentage uptake), and then calculated person-level changes in physical activity and population-level changes in distance travelled by mode.

Intervention changes in physical activity were calculated by the change in moderate and vigorous physical activity, expressed as a change in MET (metabolic equivalent of task) minutes per week of moderate and vigorous activity (MVPA-METmins/week). A MET is the ratio of work metabolic rate to a standard resting metabolic rate, where one MET is equivalent to sitting quietly [17].

For trips that switched to walking under the intervention scenarios, we assumed that the distance travelled remained constant at a 4.4km/hr walking speed (SD 0.88) [19]. We calculated the duration of the walking trip and multiplied by 3 (SD 0.06, uncorrelated with walking speed) (assigned MET value for walking [17]) to estimate the change in MVPA-METmins. For example, a 0.6km car journey switched to walking would result in a MVPA-METmin increase of 24.5 (0.6 /4.4 * 60 * 3). For trips that switched to cycling, we followed the same process but assumed a speed of 10.5km/hr (average cycling speed of those who reported cycling trips at baseline in the Household Travel Survey (calculated from reported trip distance and duration)) and a 3.5MET values corresponding to cycling at that speed derived from the Compendium of Physical Activities [17]. Trip-level changes in physical activity and mode were aggregated to estimate average population level changes in weekly physical activity and annual mode-specific distance travelled, by age, sex, and ethnicity. Change in air pollution exposure, for the whole population, was calculated from change in distance travelled by motor vehicle.

### Intervention impact

The health impacts of modelled interventions were estimated using a proportional multi-state life table model adapted from a model previously developed to evaluate health impacts of changes in diet [21]. We simulated the health impact on the adult NZ population, alive in 2011, out until death (lifetime horizon). The business-as-usual (BAU) scenario reflects continuation of current physical activity levels and transport behaviour, and best future annual percentage change estimates for future trends to 2026 (then held constant) in all-cause mortality and non-communicable disease incidence and case fatality in New Zealand. Intervention scenario changes are assumed to continue for the remainder of the modelled population’s lifetime. We applied a 3% discount rate to QALY gains and cost-offsets, in accordance with the Burden of Disease Epidemiology, Equity, and Cost-Effectiveness Protocol and international precedent (e.g. ACE-Prevention in Australia and the recommendations of a US panel of health economists) [26]. Undiscounted results are presented in the Supporting Information.

Intervention effects were captured by combining the differences in risk factor exposure between the BAU and intervention scenarios with relative risks for the association between the risk factor and disease to generate population impact fractions–essentially a percentage change in disease incidence rate. These changes in disease incidence rates flowed through the proportional life tables to change disease prevalence and then changed disease mortality (as disease case fatality was acting on a different prevalent pool of cases). Disease-specific changes in mortality and morbidity rates were summed up across diseases in each annual cycle for each sex by age by ethnicity (Māori and Non-Māori) cohort, and subtracted or added to the BAU all-cause mortality and morbidity rates in the main lifetable to estimate QALYs gained over the remainder of the cohort’s lifespan (or up to age 110 if still alive). Changes in health system costs between BAU and intervention scenarios were also calculated by the changes in the proportion of the population experiencing incidence, prevalence, and death.

#### Physical activity

To estimate the baseline distribution of physical activity, we converted responses to the New Zealand Physical Activity Questionnaire Short Form in the New Zealand Health Survey to MVPA-METmins/week. Brisk walking was assigned a MET value of 3.0 [17], and moderate and vigorous activities MET values of 4.5 and 6.5 respectively [28]. For example, an individual who reported 30mins of brisk walking per week would have 90MVPA-METminutes/week of physical activity. Observed physical activity levels were smoothed to estimate sub-population physical activity distributions by fitting a lognormal distribution separately by sex, ethnicity (Māori and Non-Māori), and age group.

#### Road injuries

Changes in road injury under the intervention scenarios were calculated proportionally from changes in distance travelled by pedestrians, cyclists, motorcyclists, and motor vehicles. We then applied mode-specific safety-in-numbers coefficients to changes in distances travelled, in line with established international methods [3, 29].

#### Air pollution

Intervention changes in air pollution were based on changes in distance travelled by motor vehicles. We used data on the proportion of fine particulate matter attributed to domestic road transport in New Zealand (11%) [30] and assumed that this component of total air pollution would change in proportion to the change in distance travelled. For example, halving distance travelled by motor vehicles would halve the fine particulate matter contributed by domestic road transport.

### Healthcare costs

Disease-specific costs were derived according to an established protocol [21, 27]. These were divided into incidence, prevalence, and mortality costs based on the timing of events (first year, subsequent years, last six months of life). Pedestrians, cyclists, motorcyclists, and motor vehicle occupant injuries were costed separately. Finally, costs were scaled to ensure consistency with total healthcare costs in New Zealand, and to avoid double counting costs attributed to individuals who may simultaneously reside in multiple disease states [27]. We present change in health system costs as 2011 NZ$ and also in 2016 US$ (derived using Consumer Price Index (CPI) and Purchasing Power Parity (PPP) adjustments) to aid international comparisons.

### Emissions

We multiplied distance travelled for each trip by emissions factors to estimate baseline and intervention annual greenhouse gas emissions. Motor vehicles were assigned emissions factors based on standardised values for New Zealand–with cars assigned an emissions factor of 0.209kgCO_2_e/km [31]. The emissions factor is based on the average emissions of the fuel required to travel a kilometre and does not include embodied emissions (i.e. emissions associated with the manufacture of vehicles), nor differences in emissions based on speed or distance travelled (e.g. higher emissions for ‘cold start’[32]). We divided the emissions value of car trips by the number of people in the vehicle, assuming the number of people was one (i.e. respondent only) and assigning the full emissions value where the number of people in the car was not explicitly stated. Pedestrian and cycling trips were assigned emissions of 0.195kgCO_2_e/km and 0.094kgCO_2_e/km respectively, again reflecting the fuel (in terms of food), required to travel a kilometre. The emissions factors used for pedestrian and cycling trips assumed that energy expenditure was fully compensated with increased energy intake (in line with our assumption of interventions resulting in no change in BMI), and that the emissions profile of the food eaten to compensate had the same emissions footprint as the average New Zealand diet [16, 33].

We report changes in emissions separately for vehicular and dietary emissions to ease comparisons with previous studies that did not include the dietary emissions component. In addition, emissions changes under the intervention scenarios are given for the first year of the intervention only, owing to the incredibly wide uncertainty around future emissions factors given the pace of technological development.

### Modelling and analysis

Each scenario was simulated 2,000 times drawing probabilistically from pre-specified uncertainty distributions about each input parameter [16]. First, we ran 2,000 simulations of the individual level trip switches in R. The aggregated results from each simulation for change in physical activity and distance travelled were imported into the MSLT model, built in Excel. A custom-built Visual Basic macro was written to estimate the health impacts and cost offsets of each simulation within the Excel MSLT model, and we calculated the 2.5th and 97.5th percentiles of QALY, emissions, and cost outputs to capture uncertainty.

### Scenario analyses

We conducted scenario analyses with one risk factor switched on at a time to examine the proportion of the health gain from different components of the modelled interventions–physical activity only, road injuries only, and air pollution only. We also present results adjusted for ethnic differences in background mortality rates (see [34] for further details).

## Results

All scenarios increased the proportion of all trips made by active transport and reduced the proportion of all trips made by motorised vehicles (see Table 2). At baseline, 82% of all trips were made by motor vehicle; 12% of all car trips (as driver or passenger) were under 1km and 44% of car trips were between 1 and 5km. Switching all eligible trips under 1km to walking under scenario (a) reduced the proportion of all trips made by motorized vehicle to 79%. Under full uptake of scenario (b), only 64% of all trips were made by motorized vehicle, and the proportion of all trips made by cycling increased from 1% to 16% of all trips.

**Table 2:** Percentage of all trips made by different modes under intervention scenarios.

	Baseline	(a) switching car trips ≤1km to walking (100% uptake)	(b) switching car trips ≤1km to walking and those 1-5km to cycling (100% uptake)
Pedestrian	16	19	19
Cyclist	1	1	16
Motorbike	1	1	1
Motor vehicle	82	79	64

**Fig 2:**
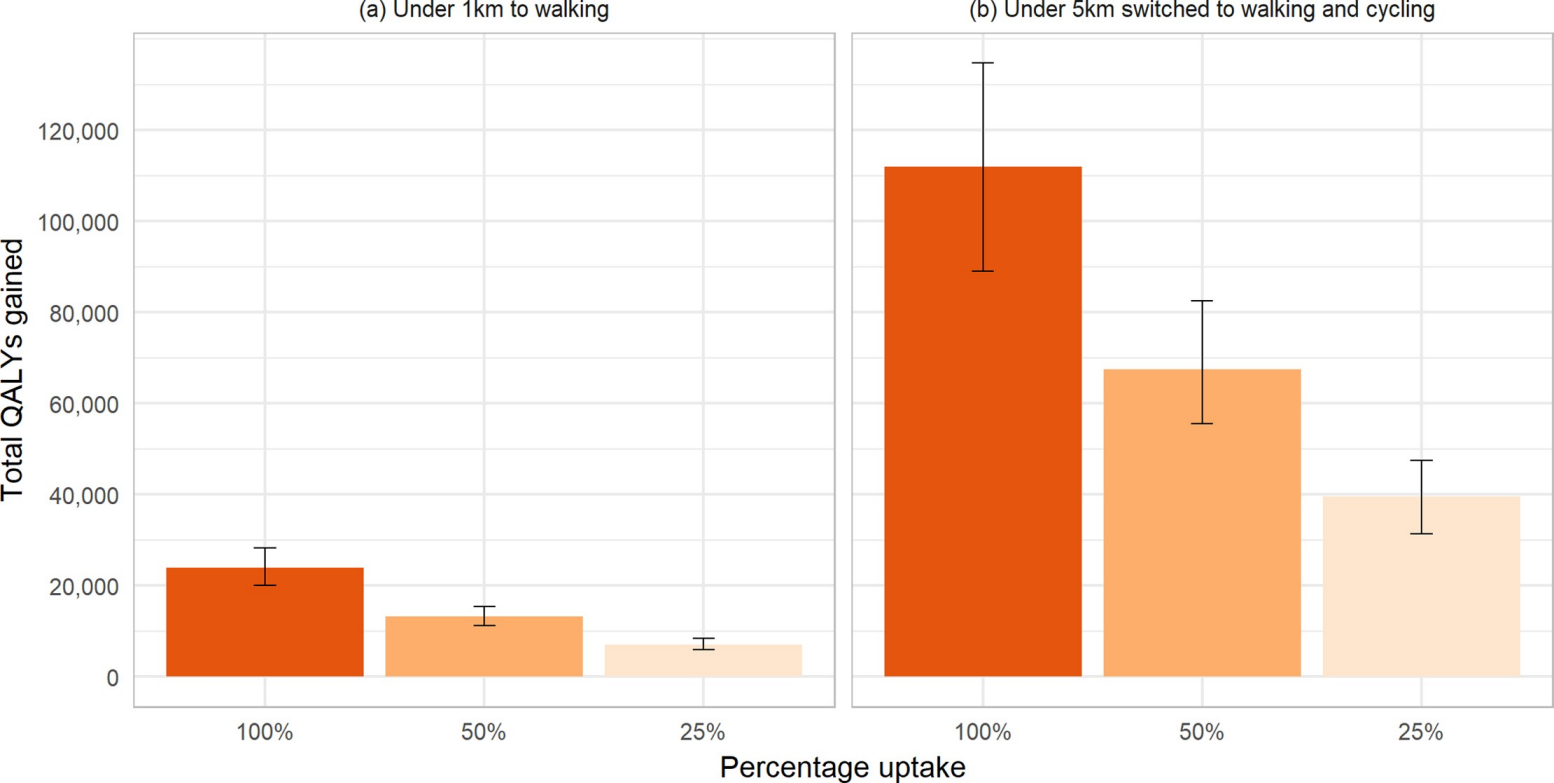
Total QALY gains from modelled interventions.

**Fig 3:**
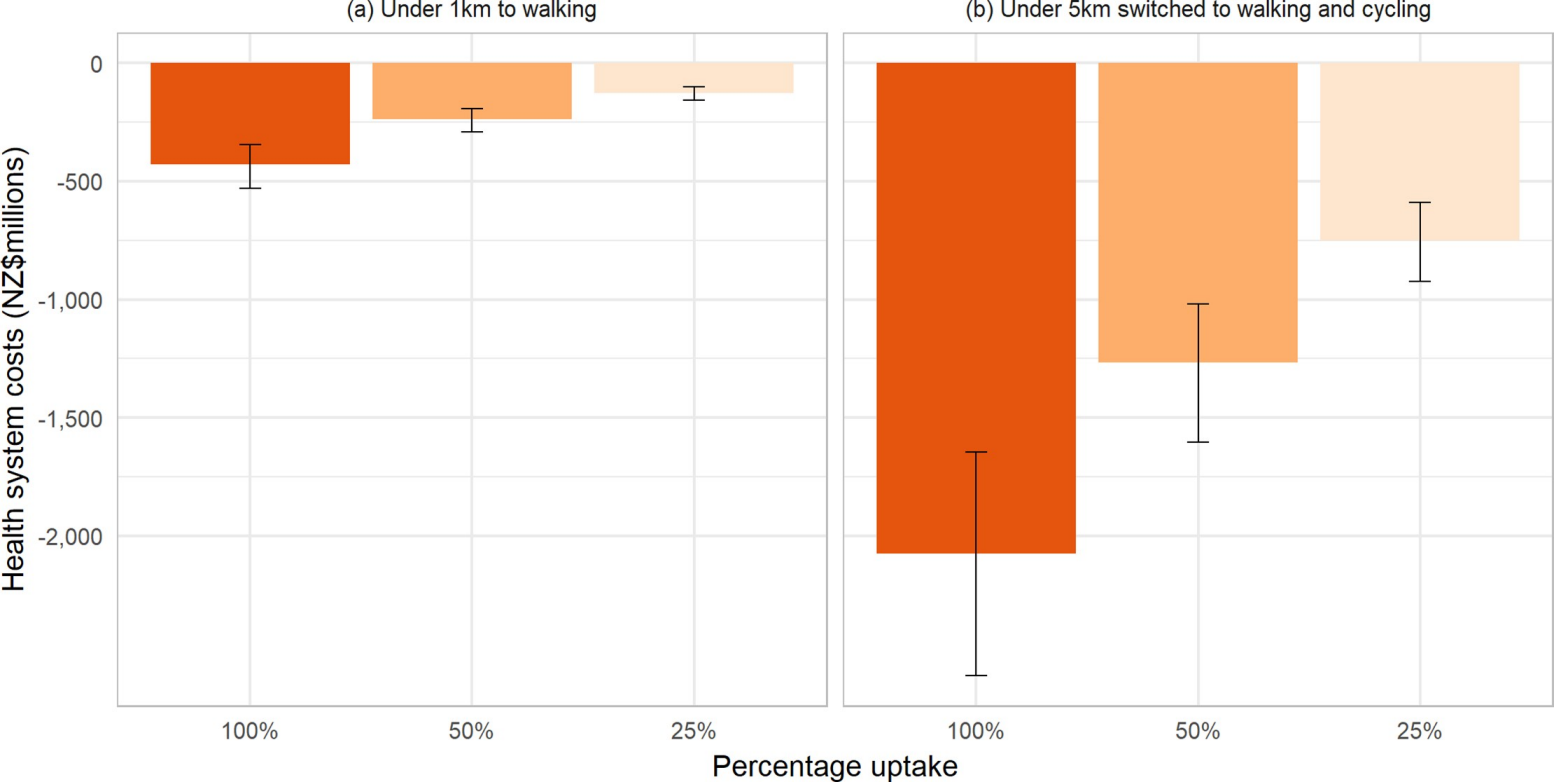
Change in health system costs from modelled interventions.

Scenario (a) resulted in 23,900 QALYs (UI 20,000 to 28,300; discounted at 3%) gained over the lifetime of the NZ population alive in 2011, and scenario (b) resulted in 112,000 QALYs (UI 89,000 to 134,700) gained under 100% uptake (see Fig 2). This equates to up to 5.42 QALYs per thousand people for scenario (a) and up to 25.43 QALYs per thousand people for scenario (b). S1 and S2 Tables display full details of the QALYs gained and change in health system costs resulting from different levels of uptake of each intervention scenario, with and without discounting. For scenario (a), 25% uptake of trip switched led to 30% of the total health gain that could be achieved if all eligible trip pairs were switched (as the relationship between PA and disease incidence is one of diminishing marginal returns for increasing PA, the percentage of trips switched and percentage of total health gain are not the same); 50% uptake accounted for 55% of the total health gain. On a per capita basis, QALY gains were generally larger in males than females, larger in Māori than Non-Māori, and largest in the 40–59 year old age group (see S3 Table). All scenarios led to reductions in health system costs (see Fig 3). These ranged from cost savings of $127million (NZD in 2011, equivalent to $90million US$ in 2016) for 25% uptake of scenario (a) to $2.1billion (NZD in 2011, $1.5billion in 2016 USD) for full uptake of scenario (b).

**Fig 4:**
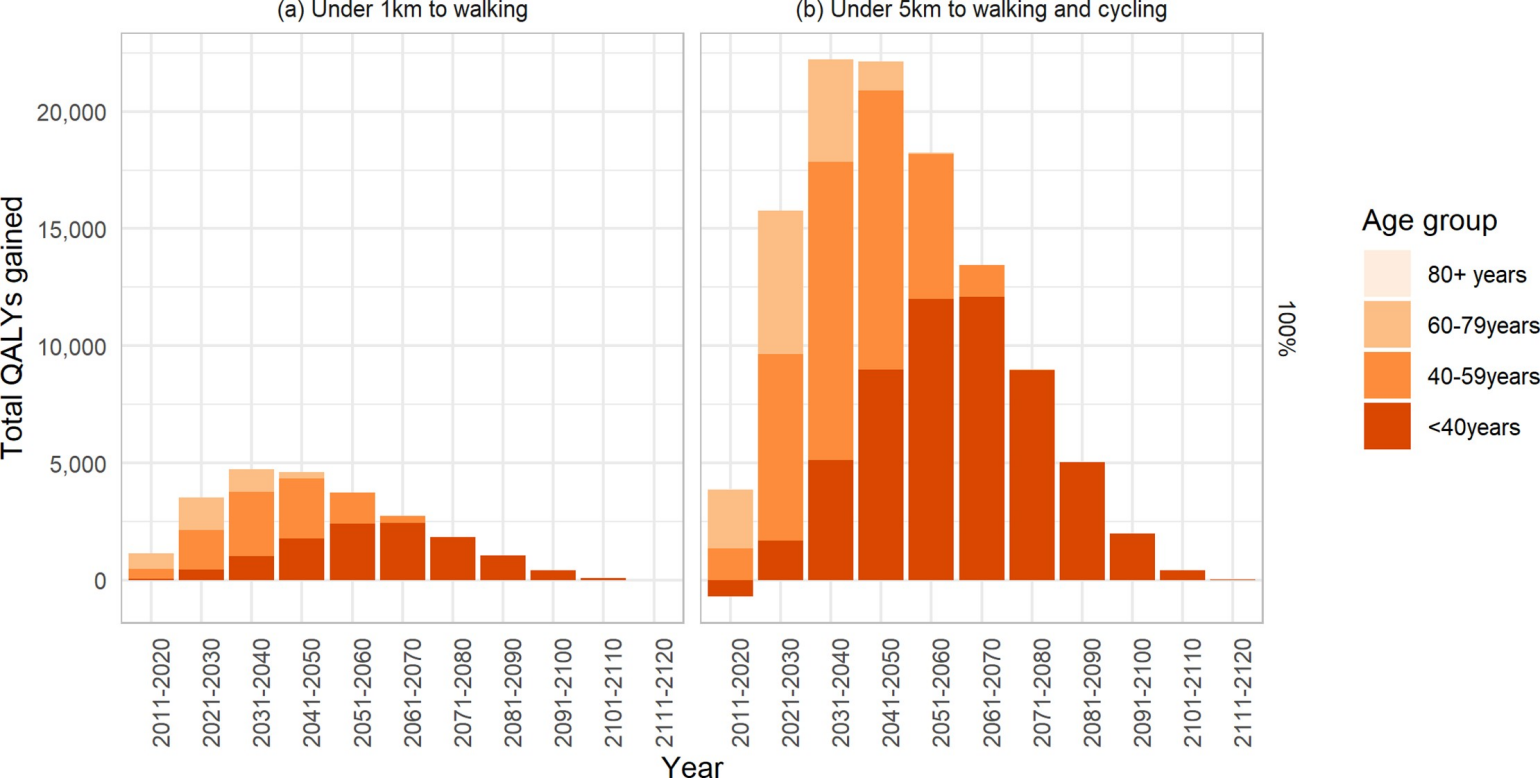
Contribution of risk factors to QALY gains under modelled interventions.

By modelling the health impact of each risk factor individually, we were able to determine that the health impacts were primarily driven by increases in physical activity (see Fig 4). All interventions led to an increase in road injuries that offset around 3% of the health gain in scenario (a) and up to 10% of the health gain in scenario (b). Under all intervention scenarios, the contribution of reduced air pollution amounted to under one percent of the QALY gains observed.

**Fig 5:**
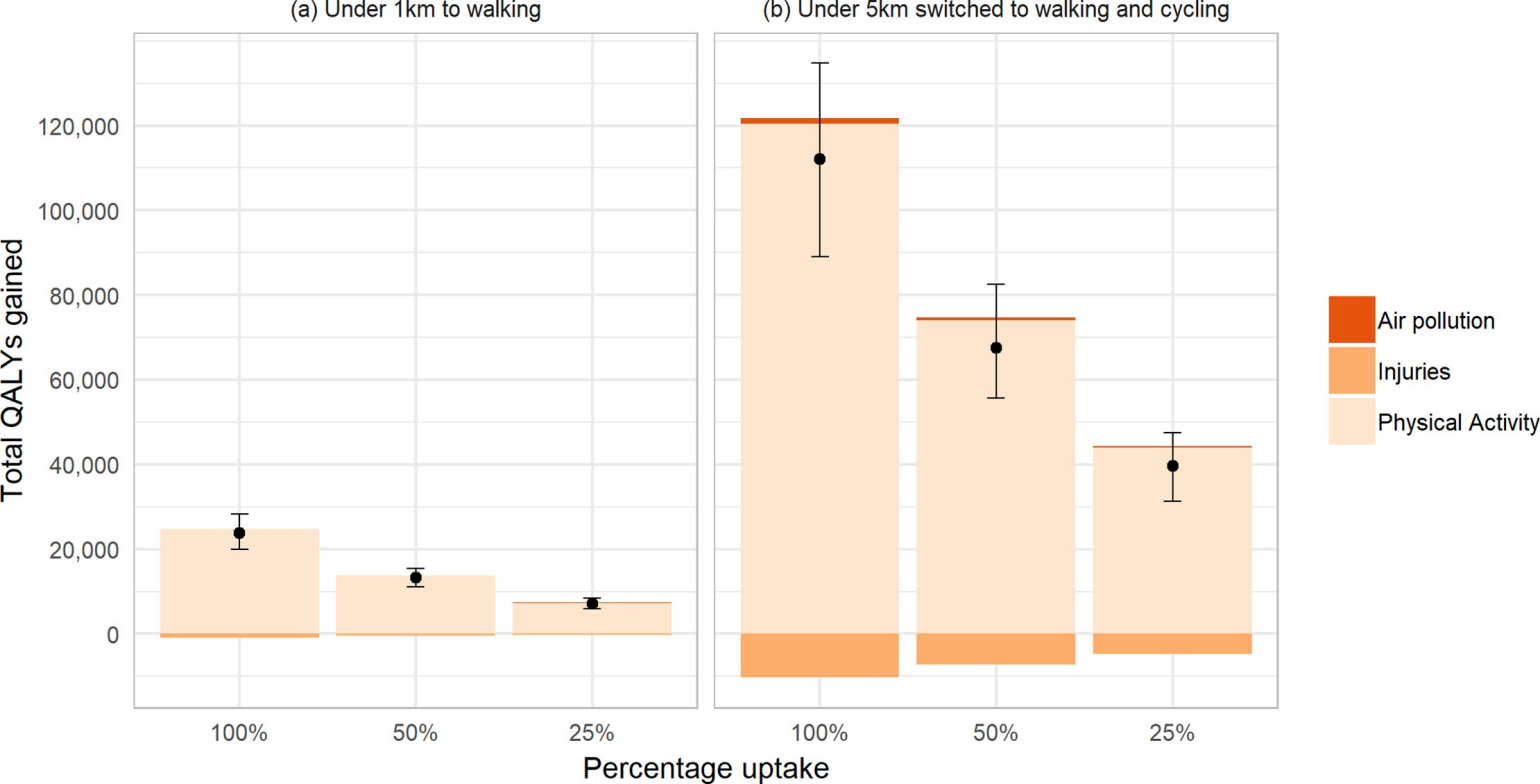
Timing of QALY gains, by age group, under 100% uptake of modelled interventions.

Although the intervention took effect immediately, the peak health gains from the modelled interventions were predicted to occur between 2031 and 2040 for the walking scenario and between 2041 and 2050 for the walking and cycling scenario (see Fig 5, and S1 Fig for <100% uptake). In the youngest age group (<40years), health gains were negative in the first ten years of the walking and cycling scenario which reflects the low incidence of non-communicable diseases relative to incidence of road injuries. Although we did not model trip switches in the 80+ age group, there were small positive health impacts under both scenarios due to reduced injuries due to the reduction in total distances travelled by motor vehicle.

There were reductions in vehicular emissions under all intervention scenarios, as displayed in Table 3. Changes in vehicular emissions ranged from -5.6ktCO_2_e/year (UI -7.8 to -3.4) for 25% uptake of scenario (a) to -436ktCO_2_e/year (UI -607.2 to– 267.6) for 100% uptake of scenario (b); corresponding to up to 4% of emissions associated with road transport in New Zealand. Reductions in vehicular emissions were compensated by increases in dietary emissions from increased energy expenditure (and therefore assumed increases in food intake) due to increased walking and cycling; for scenario (a) this led to small but insignificant increases in overall emissions. Scenario (b) resulted in significant reductions in emissions, even after allowing for increased emissions from increased dietary intake.

**Table 3:** Change in vehicular, dietary, and total greenhouse gas emissions under modelled interventions.

		Change in emissions (ktCO_2_e)		
Scenarios	Percentage uptake	Vehicular	Dietary	Total
(a) switching car trips ≤1km to walking	100%	-22.5 (-32.0 to -13.5)	24.8 (15.4 to 34.5)	2.4 (-11.1 to 15.3)
	50%	-11.3 (-15.8 to -6.9)	12.4 (7.6 to 17.5)	1.1 (-5.3 to 7.6)
	25%	-5.6 (-7.8 to -3.4)	6.1 (3.7 to 8.5)	0.5 (-2.7 to 3.8)
(b) switching car trips ≤1km to walking and those 1-5km to cycling	100%	-436.4 (-607.2 to -267.6)	241.3 (156.6 to 330.2)	-194.4 (-377.2 to -3.1)
	50%	-218.0 (-302.5 to -136.0)	121.3 (79.0 to 163.8)	-97.5 (-192.5 to -2.7)
	25%	-108.1 (-153.3 to -65.7)	60.3 (39.6 to 81.8)	-47.2 (-96.9 to -1.9)

## Discussion

Increasing active transport by switching short trips to a combination of walking and cycling resulted in positive health impacts, substantial savings in healthcare costs, and may also reduce greenhouse gas emissions. The majority of the health gains from modelled interventions were due to increases in physical activity as opposed to air pollution. This is the first study that simultaneously quantifies the health impact, health care cost savings, and changes in transport-related greenhouse gas emissions associated with switching short trips to walking and cycling at the national level.

### Comparison with previous literature

Our results support the findings of previous literature that show health benefits from increasing active transport. In line with previous research, we find that increases in physical activity account for the majority of health gains for active transport interventions and more than compensate for increases in road injuries [1–3].

Our overall results are similar to those of a recent Australian study estimating the health impacts of increasing active travel in Brisbane using a MSLT modelling approach [6]. The authors estimated per capita gains of around 28.8 health-adjusted life years per thousand (3% discounted) for an intervention that reduced the proportion of trips made by car from 82% to 63%, a similar intervention and result to the full uptake of our combined walking and cycling scenario.

The relative reduction in health gain from increased road injuries as a proportion of the total health gain of interventions that increase physical activity is also similar to previous research. Stevenson et al [35] found reductions in health gains from increased road injury to be up to 7% of total health gains observed across a range of cities worldwide; road injury increases also amount to between 2 and 20% of the total disability adjusted life years gained from interventions to increase walking and cycling in California [1]. These studies demonstrate the need for road safety improvements alongside strategies to increase active transport, to ameliorate real and perceived road safety issues associated with active transport uptake, and to maximise health gains. Strategies to improve road safety are particularly important to ensure positive shorter-term health impacts of increased active transport in younger age groups who experience low non-communicable disease prevalence but high risk of road injury.

The overall greenhouse gas emissions reductions we observe for 100% uptake of the walking and cycling scenario are equivalent to up to 64,000 people flying return between London and Auckland, or up to 1.4% of total emissions from road transport in NZ. The reductions modelled here are smaller than previous estimates as we attribute greenhouse gas emissions factors to walking and cycling (as the current study included estimates of GHG emissions associated with increased food intake), as well as to motorised vehicles. Our emissions factors for walking and cycling are based on the assumption that individuals completely compensate for the increased energy expenditure resulting from walking and cycling, and that the emissions of foods that are compensated is comparable to that of current diets in New Zealand [33]. Meta-analysis of the impact of active transport on BMI shows minimal impact [36], hence we assumed that walkers and cyclers increase food intake directly proportion to changes in energy requirements. However, if energy expenditure was not fully compensated, then the emissions associated with walking and cycling would be lower and there would be additional (likely substantial) health impacts from reduced obesity. In an Australian modelling study which assumed that increased active transport would result in reduced obesity, the change in obesity alone resulted in 80% of the health gains of all risk factor components combined (i.e. obesity, road injury, physical activity, and air pollution combined)[37].

Regarding the GHG emissions, the dietary component of greenhouse gas emissions is amenable to change–dietary greenhouse gas emissions can be reduced by 70–80% by adopting more sustainable diets (e.g. by reducing meat and dairy intake) [38]. This emphasises the need for changes towards sustainable diets regardless of, or in parallel to, increasing transport-related physical activity.

**Fig 6:**
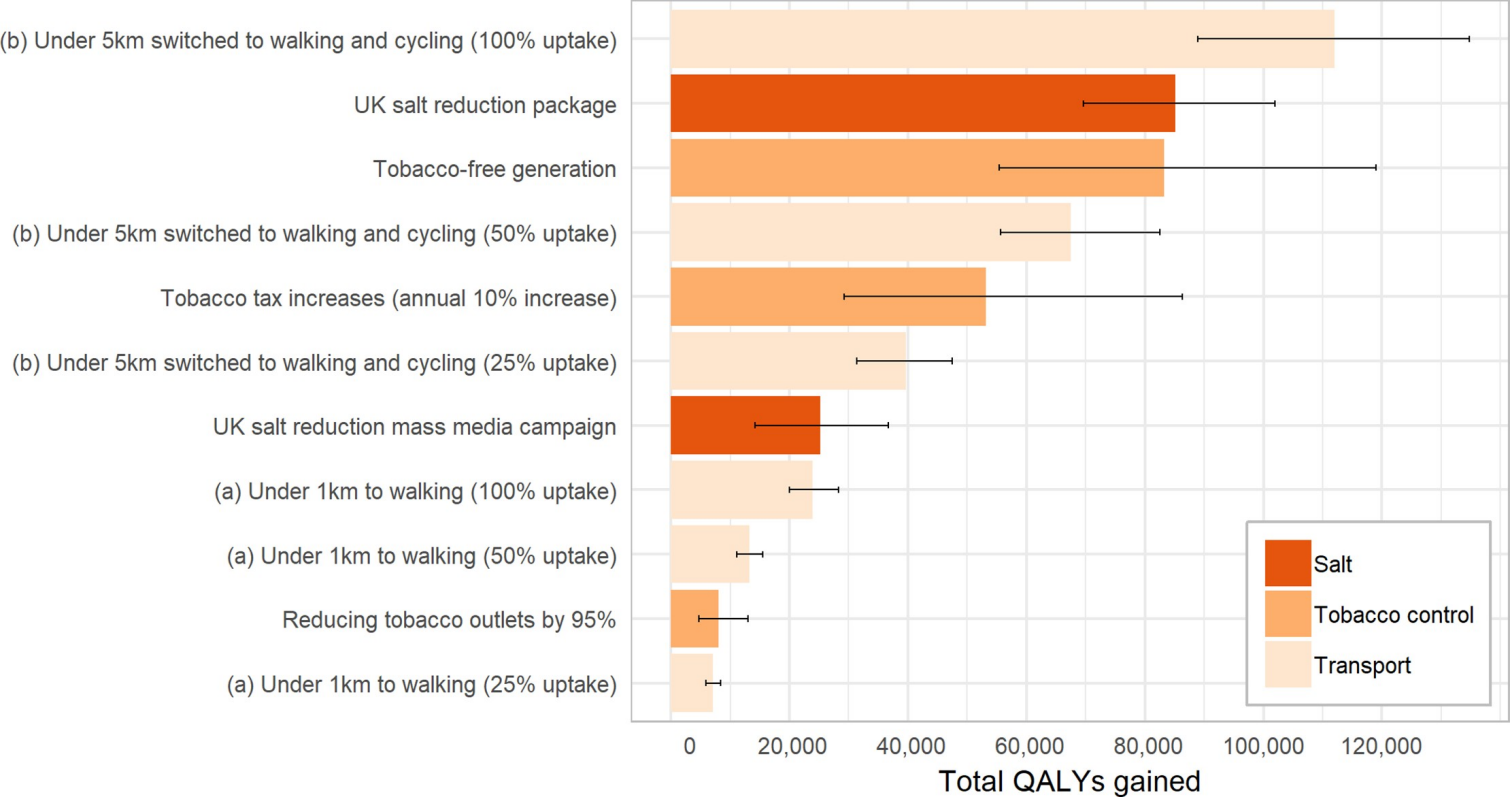
Comparison of active transport scenarios with previously modelled interventions.

The modelling framework used in this study is comparable to previous work in NZ and this allows us to compare changes in transport interventions against other public health interventions. Fig 6 plots the results of this study against previously modelled interventions for reducing tobacco [15, 39] and reducing salt in New Zealand [40]. The 25% uptake of scenario (a) had similar modelled health gains to reducing tobacco outlets by 95%, and the 100% uptake of scenario (b) had greater health impacts to the modelled health impact of a tobacco-free generation or a UK-style salt reduction campaign. That is, these are substantial health gains.

### Strengths and limitations

Our study shows the health impacts associated with switching there-and-back trips to a combination of walking and cycling, which is more plausible than just switching any short trips are made by car due to the need for the car for subsequent trips. We recognise that switching all possible trip pairs (i.e. 100% uptake) is not plausible as some will represent trips made to transport heavy goods (e.g. moving furniture) or trips made by (or with) individuals with limited mobility. However, there may be other trip combinations that are amenable to switching that were not considered as part of this analysis (e.g. A → B → C → A).

We present results for differing levels of intervention uptake and show that there are disproportionate health gains at even low levels of intervention uptake; 25% uptake of trip switches delivers around a third of the total health gain possible from switching all eligible trips. This is due to dose-response relationships between physical activity and cardiovascular diseases demonstrating decreasing marginal returns as physical activity increases [28]. However, this pattern would change if trip switching was clustered within individuals. For example, if trip switches were clustered within individuals with low levels of physical activity then the health gains would be larger.

We were not able to account for patterns of clustering of trips within households. This means that two survey respondents, in the same household, making the same sequence of trips were treated independently in our analysis. This was necessary as we were not able to identify the same trip (in the same vehicle) across multiple survey respondents, and not all people making a trip were captured in the dataset (i.e. not all individuals making a particular trip were survey respondents). This is unlikely to be problematic in the context of the hypothetical scenarios examined in this study, but may be an important consideration for future work examining more targeted behaviour change strategies. For example, for analysis of a family-targeted active transport intervention it would be important to ensure that all family members “switched” at the same time to accurately capture the health impact of the intervention.

The MSLT modelling approach adopted in this study allows us to examine the timing of health gains and account for time lags between changes in transport behaviours and reductions in disease incidence. Traditional CRA approaches overestimate the benefits of physical activity relative to MSLT modelling approaches [12], and therefore it was unclear whether the benefits of increased physical activity would continue to override the negative impacts of increases in injuries. Our study shows that it does–though injury impacts may predominate in the short term in younger age groups.

We lacked data relating transport-related physical activity behaviours to current physical activity levels. We assumed that increases in physical activity under the intervention scenarios were independent of baseline physical activity levels. This may underestimate the health impacts of the modelled interventions if those who make more of their short trips by car have lower physical activity levels, again, due to decreasing marginal returns at high levels of physical activity. We also assumed that increases in transport related physical activity did not displace other physical activity (e.g. recreational physical activity). As current physical activity levels are low, it is unlikely that increases active transport would displace other physical activity but further evidence is needed to establish the extent of substitution under specific policies.

Our estimate of the health impacts related to air pollution are based on vehicle distance travelled. This captures the overall improvement in air quality due to reduced vehicle distance. We do not capture the change in trip-level air pollution exposure for an individual who switches from a car trip (at a rest breathing rate) to an active trip involving a higher breathing rate. The impact of this could be positive (if outdoor air quality is good) or negative (if active trip involves longer exposure to areas with poor air quality due to slower speed). There was insufficient data to determine air pollution exposure at the individual level within the dataset; whilst breathing rates are higher for active modes, the quality of air breathed during the trip could be better or worse. Whilst negative impacts of increased air pollution exposure associated with breathing are unlikely to negate benefits from physical activity [41], examining real-world changes in air pollution exposure from changes in travel mode is needed.

We assumed that switching short trips to active modes would not impact on BMI, in line with findings of a recent review that argued that there were minimal changes in BMI from increases in active transport [36]. Previous modelling studies have assumed BMI reductions based on zero compensation of energy intake [1, 2], and therefore likely overestimate the health impacts of modelled interventions. However, there is emerging evidence from observational studies suggesting that individuals who transition from walking to cycling may have a decrease in BMI [42, 43]. Further research is needed to establish the extent to which additional energy expenditure from increased walking and cycling is compensated by increased food intake. Different interventions may have different BMI impacts (e.g. mass media campaign encouraging walking for weight loss compared to infrastructure improvements). Further research is also needed to determine the BMI impacts of specific active transport interventions to allow more comprehensive estimates of health and greenhouse gas emission impacts. Interventions that lead to BMI reductions in addition to physical activity level increases could have much larger health gains (and greater reductions in greenhouse gas emissions) than those presented here.

Our emissions assumptions for car journeys do not reflect systematic variation in car emissions such as higher emissions for ‘cold starts’ nor differences based on engine size. Emissions for short car trips tend to be higher on a per kilometre basis than those of longer trips due to the fuel required to warm the engine, but the magnitude of this impact is likely to be small [32]. There may also be systematic differences in the car types used for shorter trips, but there was insufficient detail on car type at the trip level to include this in our analysis. If the average engine size of cars used for short trips is larger than average then our scenarios may result in even greater reductions in vehicular emissions.

Finally, this study examines the healthcare cost implications associated with increases in active transport. Whilst we do not model intervention costs for our hypothetical intervention scenarios, the healthcare cost savings provide an indication of the cost-effectiveness threshold for interventions to improve walking and cycling. From a health system perspective, an intervention (e.g. a mass media campaign or infrastructure improvements) that results in switching 25% of trips to walking would be cost saving up to an intervention cost of NZ$127million (US$90million) and cost effective (at the $45,000 per QALY threshold) up to around NZ$445million (US$317million). Our estimate of the cost-effectiveness threshold for walking and cycling is considerably higher than the total amount spent on walking and cycling investment by the New Zealand Transport Agency between 2008 and 2017 (~NZ$120million [44]).

This study adds to the growing body of research around the impact of increasing active travel. Increases in active travel could provide a meaningful increase in physical activity at the population level, but may not be enough to address low physical activity levels alone. Interventions to encourage active transport need to address issues around road safety, but recognise that the long-term benefits of increased physical activity far outweigh road injury risks.

## Conclusions

Switching short trips to walking and cycling would have positive health impacts, reduce healthcare costs, and may also reduce greenhouse gas emissions. Further research is needed to identify viable strategies to increase uptake of walking and cycling for short trips in highly car dependent societies such as New Zealand.

## Supporting information

S1 Table Table of health gains and healthcare cost savings from modelled interventions (main result). (DOCX)

S2 Table Table of health gains and healthcare cost savings from modelled interventions (no discounting). (DOCX)

S3 Table Quality adjusted life years gained per 1,000 people by sex, age, and ethnicity. (DOCX)

S1 Fig Timing of health gains across modelled interventions. (DOCX)
