# Supplementary material for: Potential of active transport to improve health, reduce healthcare costs, and reduce greenhouse gas emissions: A modelling study
Source: PLoS One. 2019 Jul 17;14(7):e0219316. doi: 10.1371/journal.pone.0219316 (PMC6636726; doi:10.1371/journal.pone.0219316)
Supplement: S1 Table — (DOCX) [file pone.0219316.s001.docx]

**S1: Table of health gains and healthcare cost savings from modelled interventions (main result)**

| 3% Discounting | Percentage uptake | Total QALYs gained (UI) | Change in health system costs (2011 NZ$, millions) |
| --- | --- | --- | --- |
| Total |  |  |  |
| (a) switching car trips ≤1km to walking | 100% | 23,900 (20,000 to 28,300) | -430 (-529 to -345) |
|  | 50% | 13,200 (11,100 to 15,500) | -237 (-292 to -192) |
|  | 25% | 7,100 (5,900 to 8,300) | -127 (-157 to -101) |
| (b) switching car trips ≤1km to walking and those 1-5km to cycling | 100% | 112,000 (89,000 to 134,700) | -2,076 (-2,592 to -1,645) |
|  | 50% | 67,400 (55,600 to 82,500) | -1,266 (-1,604 to -1,020) |
|  | 25% | 39,600 (31,300 to 47,500) | -750 (-923 to -591) |
| Per 1,000 people |  |  |  |
| (a) switching car trips ≤1km to walking | 100% | 5.42 (4.54 to 6.42) | -0.1 (-0.12 to -0.08) |
|  | 50% | 3 (2.53 to 3.51) | -0.05 (-0.07 to -0.04) |
|  | 25% | 1.61 (1.35 to 1.89) | -0.03 (-0.04 to -0.02) |
| (b) switching car trips ≤1km to walking and those 1-5km to cycling | 100% | 25.43 (20.2 to 30.58) | -0.47 (-0.59 to -0.37) |
|  | 50% | 15.31 (12.62 to 18.73) | -0.29 (-0.36 to -0.23) |
|  | 25% | 8.99 (7.11 to 10.78) | -0.17 (-0.21 to -0.13) |
