# Supplementary material for: Potential of active transport to improve health, reduce healthcare costs, and reduce greenhouse gas emissions: A modelling study
Source: PLoS One. 2019 Jul 17;14(7):e0219316. doi: 10.1371/journal.pone.0219316 (PMC6636726; doi:10.1371/journal.pone.0219316)
Supplement: S2 Table — (DOCX) [file pone.0219316.s002.docx]

**S2: Table of health gains and healthcare cost savings from modelled interventions (no discounting)**

| 0% Discounting | Percentage uptake | Total QALYs gained | Change in health system costs (2011 NZ$, millions) |
| --- | --- | --- | --- |
| Total |  |  |  |
| (a) switching car trips ≤1km to walking | 100% | 86,600 (72,400 to 103,900) | -1,169 (-1,454 to -920) |
|  | 50% | 47,900 (39,900 to 56,900) | -645 (-796 to -515) |
|  | 25% | 25,700 (21,300 to 30,600) | -344 (-432 to -269) |
| (b) switching car trips ≤1km to walking and those 1-5km to cycling | 100% | 416,400 (339,500 to 501,200) | -5,668 (-7,129 to -4,456) |
|  | 50% | 251,800 (212,400 to 306,300) | -3,470 (-4,425 to -2,754) |
|  | 25% | 148,200 (119,600 to 176,900) | -2,058 (-2,541 to -1,605) |
| Per 1,000 people |  |  |  |
| (a) switching car trips ≤1km to walking | 100% | 19.66 (16.44 to 23.58) | -0.27 (-0.33 to -0.21) |
|  | 50% | 10.88 (9.05 to 12.92) | -0.15 (-0.18 to -0.12) |
|  | 25% | 5.83 (4.83 to 6.94) | -0.08 (-0.1 to -0.06) |
| (b) switching car trips ≤1km to walking and those 1-5km to cycling | 100% | 94.51 (77.07 to 113.77) | -1.29 (-1.62 to -1.01) |
|  | 50% | 57.16 (48.21 to 69.53) | -0.79 (-1 to -0.63) |
|  | 25% | 33.64 (27.14 to 40.15) | -0.47 (-0.58 to -0.36) |
