# Supplementary material for: Potential of active transport to improve health, reduce healthcare costs, and reduce greenhouse gas emissions: A modelling study
Source: PLoS One. 2019 Jul 17;14(7):e0219316. doi: 10.1371/journal.pone.0219316 (PMC6636726; doi:10.1371/journal.pone.0219316)
Supplement: S3 Table — (DOCX) [file pone.0219316.s003.docx]

**S3: Quality adjusted life years per 1,000 people by sex, age, and ethnicity**

|  | | | Non-Māori | | | | Māori* | | | |
| --- | --- | --- | --- | --- | --- | --- | --- | --- | --- | --- |
|  |  |  | <40 years | 40-59 years | 60-79 years | 80+ years | <40 years | 40-59 years | 60-79 years | 80+ years |
| (a) switching car trips ≤1km to walking | 100% | Males | 4.21 | 7.17 | 4.82 | 0.01 | 25.04 | 12.38 | 2.37 | 0.00 |
|  |  | Females | 4.64 | 7.21 | 5.16 | 0.01 | 10.49 | 16.48 | 9.46 | 0.01 |
|  | 50% | Males | 2.31 | 3.97 | 2.70 | 0.00 | 13.76 | 6.85 | 1.32 | 0.00 |
|  |  | Females | 2.56 | 4.01 | 2.91 | 0.00 | 5.85 | 9.21 | 5.37 | 0.00 |
|  | 25% | Males | 1.23 | 2.12 | 1.45 | 0.00 | 7.35 | 3.68 | 0.71 | 0.00 |
|  |  | Females | 1.36 | 2.15 | 1.58 | 0.00 | 3.14 | 4.95 | 2.91 | 0.00 |
| (b) switching car trips ≤1km to walking and those 1-5km to cycling | 100% | Males | 21.39 | 34.43 | 20.84 | 0.17 | 146.03 | 68.12 | 12.67 | 0.01 |
|  |  | Females | 21.61 | 32.58 | 21.56 | 0.16 | 43.36 | 66.88 | 35.72 | 0.17 |
|  | 50% | Males | 12.64 | 20.87 | 12.92 | 0.08 | 85.66 | 41.55 | 7.88 | 0.00 |
|  |  | Females | 13.05 | 20.05 | 13.63 | 0.08 | 26.07 | 40.78 | 22.36 | 0.08 |
|  | 25% | Males | 7.30 | 12.29 | 7.75 | 0.04 | 49.48 | 24.74 | 4.76 | 0.00 |
|  |  | Females | 7.64 | 11.94 | 8.32 | 0.04 | 15.04 | 23.78 | 13.40 | 0.04 |
| *Per capita QALY gains adjusted for differences in background mortality rate by ethnicity to avoid undervaluing health gains for Māori | | | | | | | | | | |
