# Supplementary figures and images for: Potential of active transport to improve health, reduce healthcare costs, and reduce greenhouse gas emissions: A modelling study
Source: PLoS One. 2019 Jul 17;14(7):e0219316. doi: 10.1371/journal.pone.0219316 (PMC6636726; doi:10.1371/journal.pone.0219316)

**S4: Timing of health gains across modelled interventions**


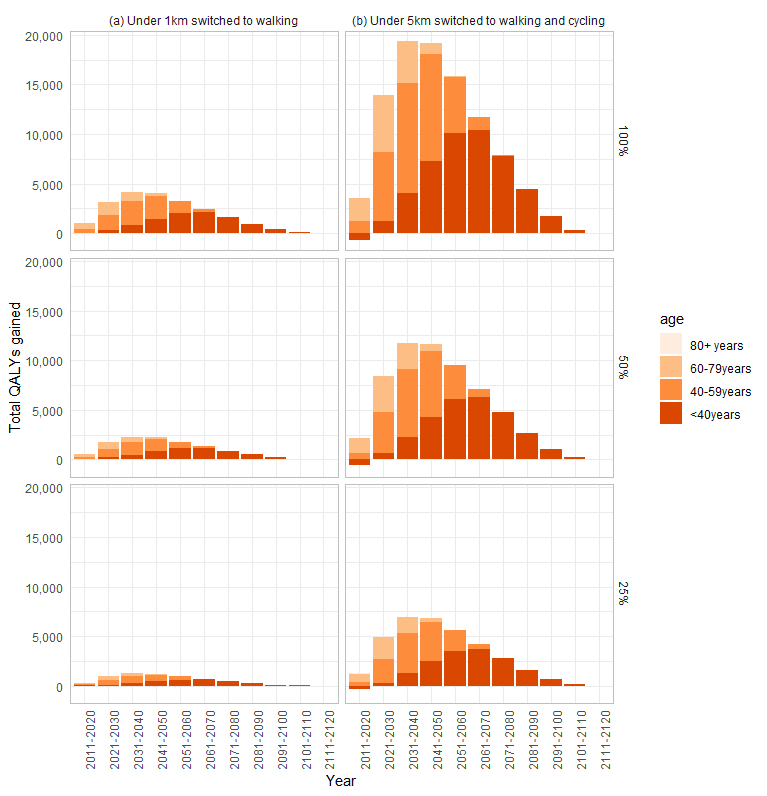

Supplement: S1 Fig — (DOCX) [file pone.0219316.s004.docx]
